# Characterization of proteins, mRNAs, and miRNAs of circulating extracellular vesicles from prostate cancer patients compared to healthy subjects

**DOI:** 10.3389/fonc.2022.895555

**Published:** 2022-12-08

**Authors:** Jolene Chisholm, Sandor Haas-Neill, Peter Margetts, Khalid Al-Nedawi

**Affiliations:** Department of Medicine, McMaster University, Hamilton, ON, Canada

**Keywords:** extracellular vesicles, proteomics, transcriptom, miRNA, mRNA, circulating extracellular vesicles

## Abstract

Prostate cancer (PC) is the fifth leading cause of death in men globally. Measurement of the blood PSA level is still considered the gold-standard biomarker test for PC despite its high rate of delivering false positives and negatives that result in an inappropriate medical response, including overtreatment. We collected extracellular vesicles (EVs) from the blood plasma of PC patients with organ-confined, extracapsular-invading, and seminal vesicle–invading tumors and from healthy subjects. We examined the protein, mRNA, and miRNA content of these EVs using mass spectrometry (MS), a human PC PCR array, and a miScript miRNA PCR array, respectively. The proteomic analysis showed distinct groups of proteins that are differently expressed in each group of patients, as well as in healthy subjects. Samples from healthy subjects and each tumor type were used for both mRNA and miRNA arrays. The mRNA analysis showed distinct groups of mRNAs that were overexpressed in healthy or in one of the three tumor types but not in the EVs of the other groups. The miRNA analysis showed distinct groups of miRNAs as well. The fold of regulation in the expression of the identified mRNA and miRNA of each stage of the disease from healthy subjects showed that various mRNAs and miRNAs could discriminate the disease stage. Overall, our data suggest many molecular marker candidates for distinguishing between healthy subjects and PC patients using the cargo of circulating vesicles, as well as markers to discriminate between the different tumor types. Once verified, these markers might have a diagnostic value for PC.

## Introduction

Prostate cancer (PC) is the fourth highest incidence cancer globally, with 1.1 million cases, and is the fifth leading cause of cancer mortality in men (375,304 deaths in 2020) (1). In 2020, PC was the second most common cancer in men worldwide, and there were 1.4 million cases of PC diagnosed (2). In approximately 70% of patients with advanced PC, bone metastasis occurs (3, 4).

Serum prostate-specific antigen (PSA) levels are considered the standard biomarker test for PC (5). Although the test has high sensitivity and, when done serially, leads to reduced mortality, it has low specificity, resulting in false positives and the mischaracterization of benign prostate hyperplasia (BPH), or any other non-cancer prostate condition as PC (6–8). A serum PSA value of 4 ng/ml or greater is widely considered the threshold for biopsy; however, 57.7% of the biopsies are negative for neoplastic growth (9, 10). Additionally, the amount of blood PSA increases with age, and men older than 50 with >4 ng/ml blood PSA have PC only 20%–30% of the time [10]. All things considered, there is an urgent need for an improved PSA test or for the identification of new markers with a better predictive value.

Extracellular vesicles (EVs) are small membrane compartments that are shed from both malignant and normal cells that contain proteins, RNAs, and DNAs (11). Originally thought to be a cellular waste removal mechanism, they are now believed to be involved in a host of intercellular signaling pathways and even the intercellular shuttling of functional cargo (12–14). We previously reported that EVs in the blood of PC patients contain diagnostic markers for the disease and, therefore, have important potential in PC diagnosis (15). Duijvesz et al. (16) also referred to EVs as biomarker treasure chests for PC.

Free-circulating proteins are the biomarkers most widely used across multiple cancers (17). In recent years, however, circulating EV biomolecules (miRNA, mRNA, and proteins) have increased in popularity because they can be isolated from blood prior to analysis. This effectively increases the measuring instrument’s capability (18–21). For PC specifically, EVs collected from urine and semen have also been considered worthy biomarker containers (22, 23). Of the three body fluids, semen is considered rich in prostate-derived EVs; however, its collection can prove challenging in older men, particularly those with prostate disease, defeating the purpose (24). Urine is the second source for prostate-derived EVs; however, its consistency is highly dependent on a patient’s nutrition and hydration levels (23). It is for these reasons that circulating prostate-derived EVs offer the most consistent molecular markers for the early detection and staging of PCa.

miRNAs are small non-coding RNAs that average 22 nucleotides in length. They are involved in a variety of functions including the suppression of translation or mRNA degradation through the RNAi pathway (25–27). miRNA expression has also been shown to be regulated differently in cancer in ways that facilitate cell proliferation and resist cell death (28–31). Many studies have suggested the use of miRNAs as biomarkers for the diagnosis and the determination of the prognosis in cancer since circulating miRNA levels may reflect the state of the tumor (32).

mRNA has promise as an indicator of the PC outcome and the examination of its expression in PC tumor tissue can be shown to predict lethal and metastatic cancer progression in men with localized PC (33). When expression profiles from miRNA and mRNA arrays for tumor tissue are combined, they are sufficient to differentiate tumor tissue from benign (34). EVs are very stable and protective of their nucleic acid content (35). PC cell-line EVs have been shown to have significantly different mRNA contents than their healthy counterparts, but little work has been done to show the potential for differences between the contents of EVs collected from healthy and PC patient blood plasma (36).

Herein, we examined the contents of EVs extracted from patient blood plasma and compared data on their proteomics, mRNA, and miRNA profiles to generate a library of potential markers for PC diagnosis and prognosis. These libraries of data were generated from four groups of six subjects for each group for the proteomic studies and three subjects in each group for mRNA and miRNA microarray studies. The groups are one healthy group, an organ-confined PC (OC) group, an extracapsular-extending PC (EC) group, and a seminal vesicle–invading PC (SI) group. Our data show that blood plasma EV cargo can be identified to distinguish between a PC patient and a healthy subject of the same age and can also be used to discriminate between PC patients at different stages of the disease. Upon further validation, EVs could provide simple, non-invasive, high-specificity markers to diagnose PC and monitor its progression.

## Materials and methods

### Prostate cancer and healthy subjects

Four groups of six subjects were used for the proteomics study, and another four groups were used for miRNA and mRNA studies with three subjects in each group; the four groups included one healthy group and one group for each of three different PC types. These three tumor types include organ-confined tumors (OC), tumors exhibiting extracapsular extension (EC), and tumors exhibiting seminal vesicle invasion (SI). The plasma samples for all patients have been obtained before the prostatectomy procedure, chemotherapy, or radiation treatments. The plasma samples were supplied in 1 ml/vials, previously frozen in liquid N_2_, and shipped on dry ice. Upon receiving the samples, they were kept in liquid N_2_ and thawed at the time of EV collection. Plasma collection was performed using lavender-topped K2EDTA tubes. Tumor subject samples, accompanied by full clinical and demographic information, were obtained, thanks to the Ontario Institute of Cancer Research tumor bank. The age range for the patients is (60–64) and (65–69) (the patient’s age has been supplied by the OICR tumor bank as a range of 5 years to preserve the patient’s privacy). The other demographic information includes the patient’s sex. In this study, all the patients are men, and, as for the vital status, all patients were alive at the time of receiving the samples. Healthy subject samples were obtained, thanks to St. Joseph’s Healthcare Hamilton, Ontario, Canada. The subjects were chosen with matching age to the patients and with no previous record of any type of cancer or major health concern. This study received ethical approval from the Hamilton Integrated Research Ethics board.

### Collection of extracellular vesicles from blood plasma

Differential centrifugation, as described in (13–15, 37), was used to purify EVs from plasma. In brief, 1 ml of plasma was diluted 1:1 in PBS to reduce viscosity and subjected to differential centrifugation at 2,000 *xg* for 10 min at 4°C, 12,000 *xg* for 20 mins at 4°C, and 100,000 *xg* for 2 hours at 4°C. Pelleted EVs were collected from ultracentrifuge tubes and suspended in 200 µl of phosphate-buffered saline (PBS).

### Nanoparticle analysis

EVs collected from PC patients and healthy subjects were subjected to nanoparticle analysis using the NanoSight LM14C with an infusion rate of 80 and after being diluted 1:10.

### Proteomics sample preparation

A 10% SDS-PAGE gel was loaded and run with 100 µg of vesicle protein from 1 ml of blood plasma from each subject sample and stained with Coomassie blue (BioRad). After visualization, sample bands were excised from the gel and subjected to mass spectrometry (MS)-based proteomics. Excised bands were dehydrated in 50% acetonitrile (ACN) and reconstituted in 50 mM ammonium bicarbonate containing 10 mM Tris-2-carboxyethyl phosphine before being vortexed at 37°C for 1 h. Sample alkylation was achieved with chloroacetamide at a final concentration of 55 mM and 1 µl of trypsin to perform digestion. Peptides were extracted in 90% ACN and, after a number of steps, were subjected to label-free quantification MS experiments using collision-induced dissociation in a linear ion trap. PEAKS software (Bioinformatics Solutions Inc.) was used to convert MS data to peaks lists and then to fit the data to the Human1302S database assuming trypsin to be the digestion enzyme to perform MS/MS spectra analysis. A parent ion mass tolerance of 10.0 PPM (monoisotopic) and a fragment ion mass tolerance of 0.0100 Da (monoisotopic) were permitted during the database search. The variable modifications: the deamination of asparagine and glutamine; the oxidation of methionine; carbamidomethylation of cysteine; and the phosphorylation of serine, threonine, and tyrosine were all specified in PEAKS software. The protein scored and expectation values were used to determine protein match probabilities, and resolved protein identities were considered to be correct when it contained four unique peptides and had a score higher than the identity threshold at p < 0.05.

### RNA isolation from blood plasma extracellular vesicles

An miRNeasy Minikit (Qiagen) was used to isolate RNA from vesicle suspensions containing 10 µg/µl RNA-grade glycogen (Thermo Scientific). Isolated RNA received 290 µl of ice-cold ethanol and was incubated overnight at -20°C to concentrate it. RNA was subjected to centrifugation at 10,000 rpm for 15 min and was rinsed twice in ice-cold 75% ethanol before the pellet was air-dried for 5 min and resuspended in 8 µl of RNAse-free water. Nanovue (GE Healthcare Lifesciences) was used to determine RNA concentration and quality.

### miRNA arrays

A miScript II RT kit (Qiagen) was used to perform reverse transcription. The preamplification of miRNAs prior to quantification was required to accurately assess their expression because of their low abundance in EVs. A miScript Preamp PCR kit (Qiagen) in conjunction with the miScript Human Prostate Cancer PreAMP PCR primer mix was used to perform multiplex PCR-based preamplification reactions. miScript miRNA PCR array with a Human Prostate Cancer Panel (Qiagen) was used to measure miRNA expression profiles and qPCR reactions were performed on the ABI ViiA 7 (Applied Biosystems). Six control small nuclear RNAs: SNORD61, SNORD68, SNORD72, SNORD95, SNORD96A, and RNU6B/RNU6-2 were used to normalize the expression values of miRNAs, and differential expression was calculated using the ΔΔCt method. Raw data were analyzed using the miScript miRNA PCR Array Data Analysis Website (https://geneglobe.qiagen.com/us/analyze).

### mRNA arrays

An RT^2^-Profiler PreAmp cDNA Synthesis Kit (Qiagen) was used to perform first-strand synthesis, and the resulting cDNA templates were preamplified with RT^2^ PreAMP cDNA Synthesis Primer, Human Prostate Cancer panel (Qiagen). The RT^2^ Human Prostate Cancer PCR array (Qiagen) measured the mRNA expression profiles and five endogenous controls—ACTB, B2M, GAPDH, HPRT1, and RPL–0—were used to normalize expression values. The ΔΔCt method was used to calculate differential expression, and raw data were analyzed using the RT^2^ Profiler PCR Array Data Analysis Website (https://geneglobe.qiagen.com/us/analyze).

## Results

### Nanoparticle analysis

**Figure 1:**
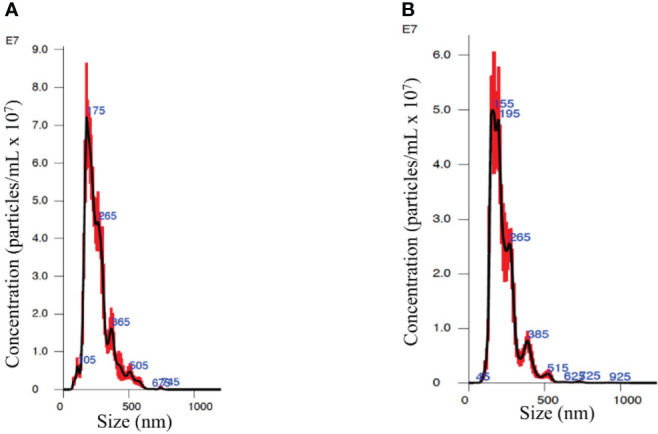
Nanosight analysis of the extracellular vesicles (EVs) from an organ-confined (OC) patient **(A)** and a healthy subject **(B)** showing similar spectrums of vesicle size and quantity.

The NanoSight showed that EVs from the plasma of both PC patients and healthy subjects (**Figures 1A, B**, respectively) are a homogenous population with an average size range between 100 and 200 nm.

### Proteomics

**Figure 2:**
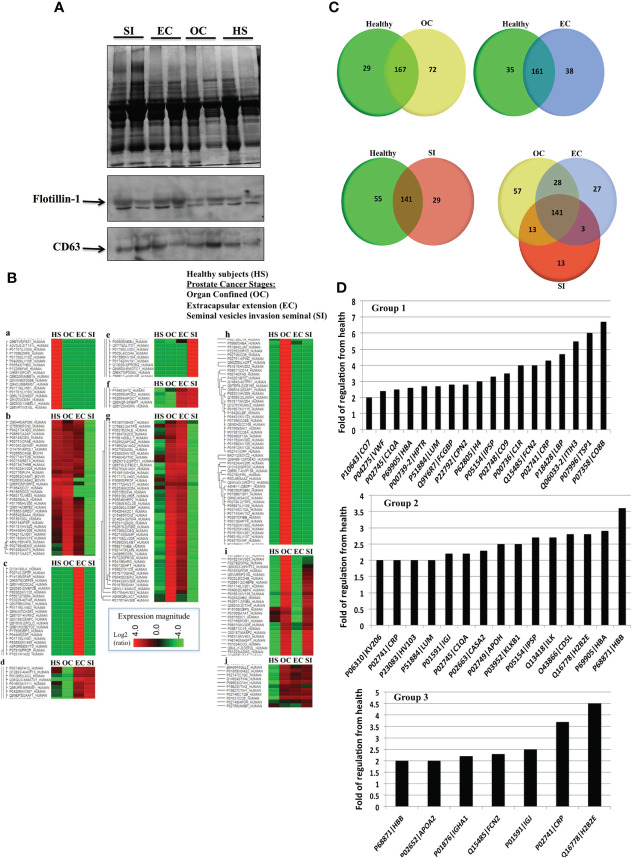
**(A)** A representative Coomassie blue stain SDS-PAGE gel for EVs collected from the plasma of prostate cancer (PC) patients in three stages of the disease and healthy subjects. **(B)** Proteomic analysis from the blood plasma vesicles of six healthy male plasma samples, six OC patient plasma samples, six extracapsular-extension (EC) patient plasma samples, and six seminal vesicle invasion (SI) patient plasma samples. There were 100 µg of protein from vesicles used for each group. The heat map shows a distinctive protein profile for each stage of the disease compared to healthy subjects. Ten clusters of proteins (a-j) were identified that could discriminate between PC and healthy subjects, and distinguish the disease stage. Figure shows in principal that EVs proteins could be used as biomarkers for each stage of tumor and relay diagnostic and prognostic information. **(C)** Venn diagrams illustrating the number of proteins unique to each tumor type group compared to healthy vesicle protein expression (a, b, c), and an expression common and unique to each tumor type (d). **(D)** Proteins with the highest folds of change from healthy EVs in the EVs of OC patients (Group 1), EC patients (Group 2), and SI patients (Group 3). Each of these values included in the graphs was found to be statistically significant (p < 0.01) by a Fisher test.

EVs were collected from the blood plasma of PC patients in three tumor types with different progression stages, i.e., organ-confined (OC), extracapsular-extension (EC), and tumor-exhibiting seminal vesicle invasion (SI) with six samples for each stage. Additionally, EVs were collected from the blood plasma of six healthy men with matching age (50–65) and had no previous history of cancer or benign tumor. **Figure 2A** shows a representative image of an SDS-PAGE gel for EVs collected from PC patients. Western blots for Flotillin-1 and CD63 were used as a marker for EVs. Proteomics analysis for all the groups is demonstrated by the expression heatmap (**Figure 2B**); the protein expression magnitude is shown as a ratio of log2 (+4 to -4). The heatmap has been divided to 10 groups of protein clusters to show the acquired protein expression patterns, which are as follows: proteins that are overexpressed in the blood EVs of healthy subjects but less so in the EVs of the patients of all tumor types (a). Proteins have increased expression in all groups but SI, which shows the downregulation of these proteins (b). Proteins are upregulated in EC patients but are downregulated in all other groups (c). Proteins are upregulated in EC and SI patients but are mildly downregulated in healthy subjects and moderately downregulated in OC patients (d). Proteins are upregulated in SI patients but downregulated in all other groups (e). Proteins are upregulated in EC and SI patients but downregulated (to a larger degree than group d) in healthy subjects and OC patients (f). Proteins are upregulated in OC and EC patients but downregulated in SI patients and, to a lesser degree, downregulated in healthy subjects (g). Proteins are upregulated in OC patients but downregulated in all other groups (h). Proteins are upregulated in OC and SI patients but largely downregulated in healthy subjects and EC patients (i). Proteins follow the opposite pattern to group a in that they are upregulated in the patients of all tumor types and downregulated in healthy subjects (j).

To further illustrate protein differentiation, we have created Venn diagrams that show the number of proteins that are unique to each subject type and the ones that are shared by multiple subject types (**Figure 2C**). When comparing vesicle (EV) proteins from healthy subjects and OC patients, there are 72 proteins expressed uniquely in the patients and 29 expressed in the healthy subjects, with 167 of the analyzed proteins being common to both. When vesicle proteins from patients with different types of PC were compared, we detected 58 proteins expressed uniquely in OC patients, 27 expressed in EC patients, 13 expressed in SI patients, and 141 common to all cancer types.

We performed a fold of regulation analysis for proteins from each tumor stage vs. control (**Figure 2D**). In organ-confined PC, these are interalpha-trypsin inhibitor heavy chain 3 (ITIH3), thrombospondin-1 (TSP1), and the compliment component C8 beta chain (CO8B) (**Figure 2D**- Group 1). In EC, these are histone H2B type 2-E (H2B2E), CD5 antigen-like (CD5L), and integrin-linked protein kinase (ILK) (**Figure 2D**- Group 2). In SI PC patients, the vesicle proteins most differentially expressed from healthy subject EVs include C-reactive protein (CRP), and histone H2B type 2-E (H2B2E) (**Figure 2D**- Group 3).

**Figure 3:**
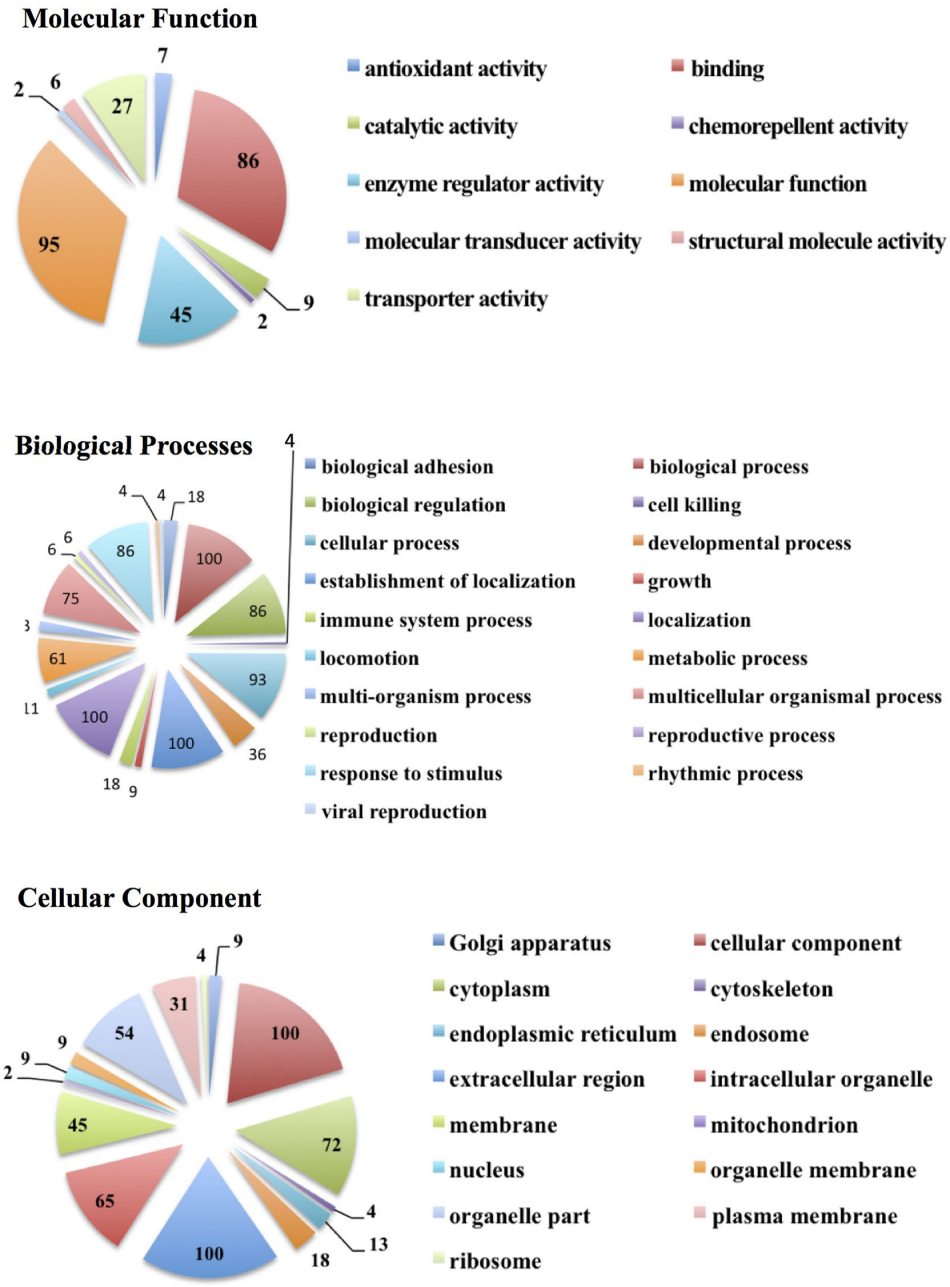
Protein ontology analysis showing the number of EV proteins from all subject groups and categorized according to molecular functions, biological processes, and cellular components.

We also carried out a protein ontology analysis of protein function, localization preference, and the biological processes associated with all proteins detected across all samples (**Figure 3**). The three most common processes these vesicle proteins were involved in were general binding (86 proteins), enzyme regulator activities (45 proteins), and miscellaneous molecular functions (95 proteins). The most common biological processes were proteins involved in general biological processes, proteins involved in the establishment of localization, and proteins involved in localization. A total of 100 proteins each belonged to these three biological process groups and comprise 36% of proteins’ biological process identified by the software. As for the cellular component, 100 proteins preferentially localize to a miscellaneous cellular component: 100 preferentially from the extracellular region and 72 preferentially from the cytoplasm. These three components make up just over 50% of all the detected proteins across all samples.

### mRNA analysis

**Figure 4:**
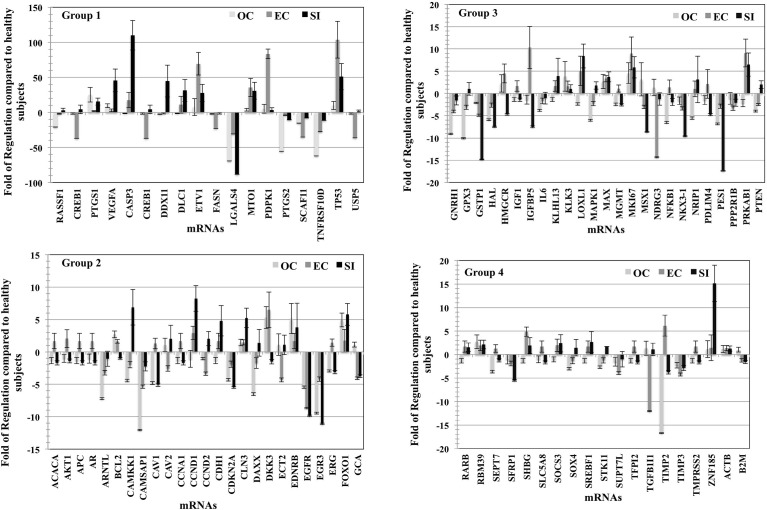
mRNA profile of plasma vesicles showing the folds of regulation in expression of specific mRNAs compared to healthy subjects. Three plasma samples from each of the three PC tumor types (OC, EC, and SI) were compared to three healthy subjects, and the results were normalized to a set of control mRNAs included in the microarray. The four groups (histograms) have various regulation scales to differentiate between mRNAs that are moderately differentially expressed to those that are greatly differentially expressed. The p-values are calculated based on a Student’s t-test of the replicate 2^(- Delta Ct) values for each gene in the control group and tumor groups. Fold regulation values greater than one indicate positive or upregulation. The fold of regulation values less than one indicate negative or downregulation; the results were considered significant at p < 0.05; the results were presented as the mean ± SD.

The fold of the regulation of expression for the detected transcripts in tumor types compared to healthy subjects was calculated and plotted (**Figure 4**); for better visualization, we used four blots according to the fold of regulation from healthy subjects for the three PC groups. mRNAs for several genes such as CASP3, DDX11, DLC1, ETV1, PTGS1, TP53, and VEGF were differently overexpressed compared to healthy subjects at an average of 25–120 fold. The mRNAs of other genes such as CREB1, FASN, LGALS4, PTGS1, SCAF11, TNFRSF10D, and USP5 were differentially downregulated in EVs from the three stages of PC patients compared to healthy subjects at an average of -20 to -90 fold (**Figure 4**-Group1). Other groups of mRNA are differentially regulated with the fold of regulation from healthy subjects ranging from (+15) to (-15) (**Figure 4**- Group 2), and (+20) to (-20) (**Figure 4**, Group 3, and 4). The characterized mRNAs translate to proteins that are involved in many aspects of cancer progression and metastasis. Taken together, these data suggest that the mRNA cargo of blood plasma EVs, like the protein, has a diagnostic value for PC, as well as the potential to differentiate between tumor types. The p-values are calculated based on a Student’s t-test of the replicate 2^(- Delta Ct) values for each gene in the control group and tumor groups. Fold regulation values greater than one indicate positive or an upregulation. The fold of regulation values less than one indicate negative or downregulation; the results were considered significant at p<0.05. The expression of these mRNAs is significantly correlated with the stage of the disease as tested by the Pearson correlation test (p<0.01).

### miRNA analysis

**Figure 5:**
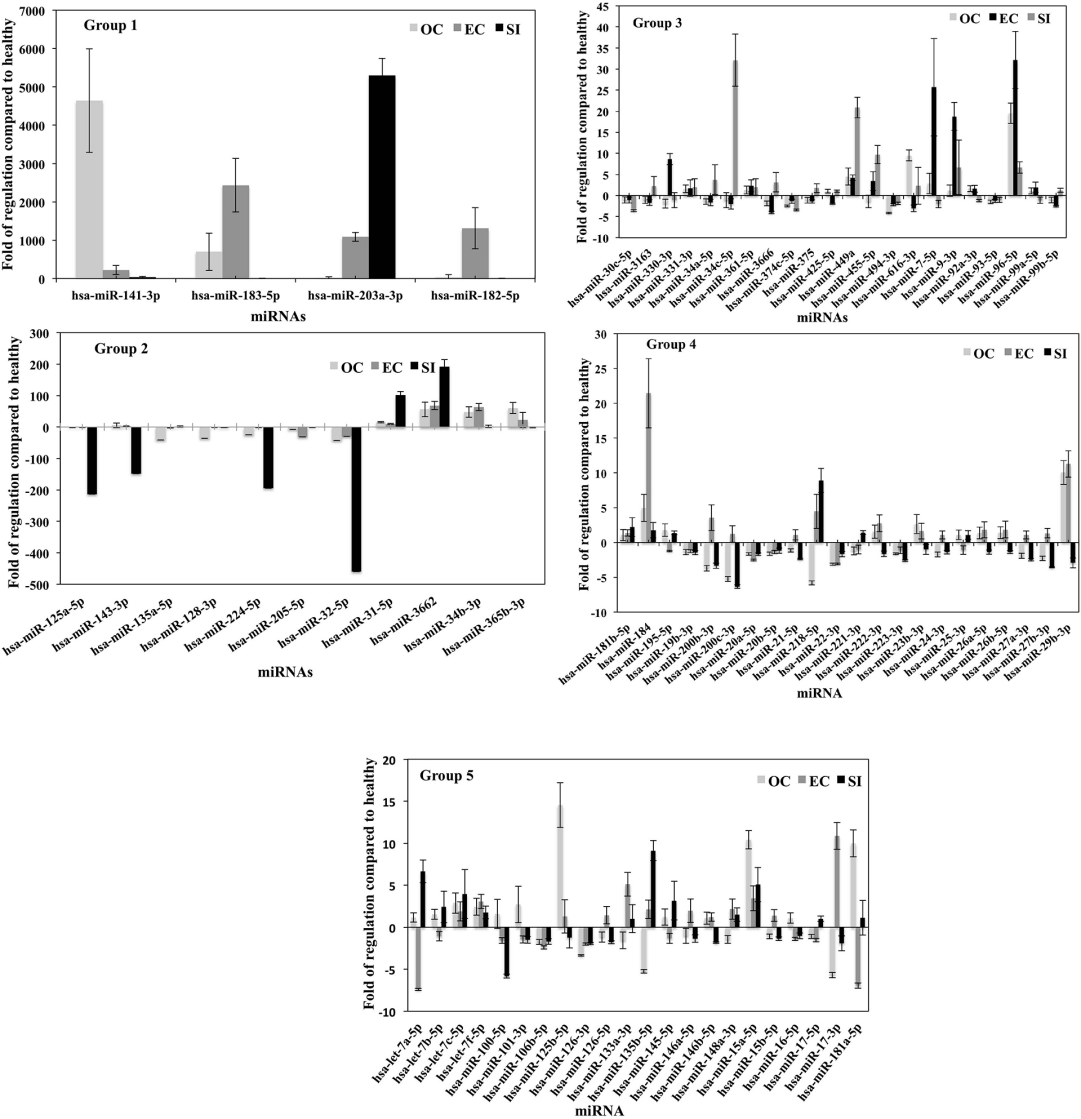
miRNA profile of plasma vesicles showing the folds of regulation in the expression of specific miRNAs compared to healthy subjects. Three plasma samples from each of the three PC tumor types (OC, EC, and SI) were compared to three healthy subjects, and the results were normalized to a set of control miRNAs included in the microarray. The five groups (histograms) have various regulation scales to differentiate between miRNAs that are moderately differentially expressed to those that are greatly differentially expressed. P-values were calculated based on a Student’s t-test of the replicate 2^(- Delta Ct) values for each gene in the control group and treatment groups, and p < 0.05 was used to consider the fold of regulation to be significant. The results were presented as the mean ± SD.

**Figure 6:**
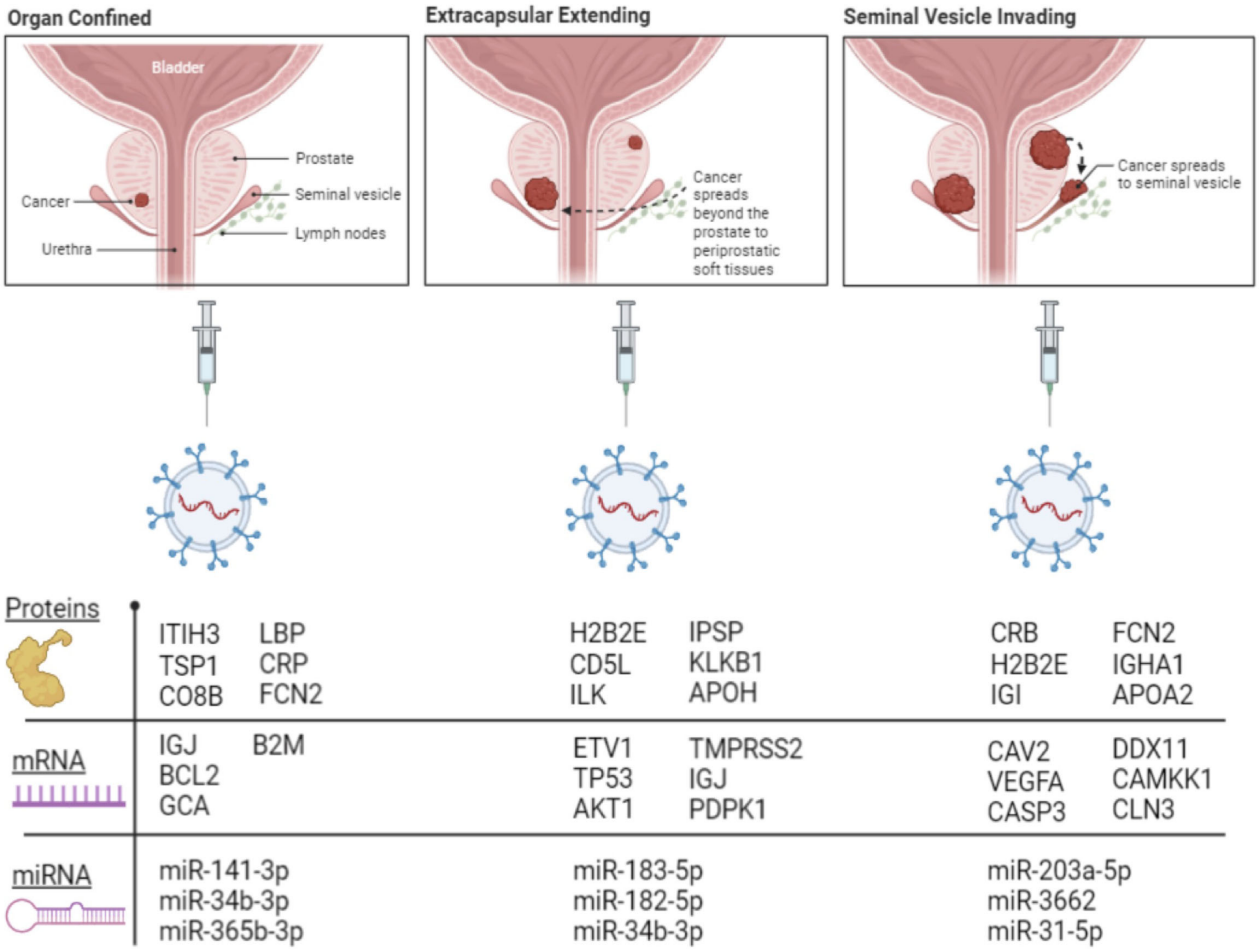
Illustration showing selected proteins, mRNAs, and miRNAs with the highest folds of change in each group of PC patients.

The fold of the regulation of the detected miRNA from each group of PC patients compared to healthy patients is calculated (**Figure 5**). The figure was divided into five histograms to accommodate the expression magnitude of the miRNAs included in the array for better visualization. The profile of the EVs-miRNAs of PC patients in stage OC, EC, and SI compared to healthy subjects showed a potential to differentiate PC patients’ stages from healthy patients and among the tumor stages. The most distinctive miRNAs separate each group of PC patients from healthy subjects represented by miRNA hsa-miR-141-3p, which has the highest degree in the fold of regulation in the OC tumor group. miR-141-3p has more than 4,000 folds of regulation compared to healthy subjects; the expression is the highest among the tumor group. Interestingly, hsa-miR-183-5p and hsa-miR-182-5p have the highest fold of expression in the EC patients with the fold of expression of approximately 2,500 and 1,500, respectively. It seems that hsa-miR-203a-3p is expressed in the SI patients group with the highest fold of expression of approximately 5,500. These four miRNAs have a specific high level of expression distinctive to each group of the tumor types. Other miRNAs were expressed differently in each group of patients with a different fold of expression but with less expression fold than the mentioned four miRNAs. Other miRNAs showed the high levels of differential expression in the studied tumor types with the fold of regulations ranging from (+300) to (-500) (**Figure 5**- Group-2). miRNAs such as hsa-miR-125a-5p, hsa-miR-143-3P, hsa-miR-224-5P, and hsa-miR-32-5p downregulation and miRNAs; hsa-miR-31-5p and hsa-miR-3662 overexpression in patients with the SI tumor indicates a potential to separate this group of patients from other tumor groups. Other miRNAs were detected to be differentially expressed with a different fold of regulations from the healthy subjects; a group of miRNAs with expression ranging from (+35) to (-5) (**Figure 5** group 3), (+25) to (-7), (**Figure 5**-group 4), and (+15) to (-7) (**Figure 5** Group 5). P-values were calculated based on a Student’s t-test of the replicate 2^(- Delta Ct) values for each gene in the control group and treatment groups, and p-values less than 0.05 were used to consider the fold of regulation to be significant. The expression of these miRNA is significantly correlated with the stage of the disease as tested by the Pearson correlation test (p<0.01). **Figure 6** summarizes proteins, mRNAs, and miRNAs with the highest folds of change for each group of PC patients. These molecules will be further validated as candidates to characterize each patient’s group from the other.

## Discussion

This research highlights the potential for EVs to be collected from the blood as marker-containing capsules for both the diagnosis of PC and for making a determination of the patient’s stage and prognosis. Each type of molecular cargo studied—protein, mRNA, and miRNA—was able to discriminate between healthy subjects and cancer patients and between tumor types. These potential EV biomarkers, after verification, could well assist in PC detection with a lower incidence of false-positive and -negative cases. PSA is said to have the ability to ‘detect metastases’ in the sense that detecting it in abundance well over 4 ng/ml suggests that it is more likely that the patient’s cancer has metastasized to the bone (5). A higher PSA level as a test for metastasis is poor because it relies on arbitrary numerical values in PSA measurements, rather than as markers that actually reflect the biological change occurring within the tumor. There are, in fact, a number of cancer-unrelated conditions and circumstances that are known to cause an abundance of blood PSA, including prostatitis, urinary tract infections, and BPH, as mentioned previously (6, 38, 39). While each of these biomarkers is not exclusively associated with cancer, elucidating a profile of vesicular biomarkers offers the most detailed picture of the unique problem. The fact that some of these EV proteins (e.g., PBIP1 and APOC2), mRNAs (e.g., DLC1 and MTO1), and miRNA (e.g., hsa-miR-99a-5p and hsa-mir-203a-3p) are present in the EVs of EC or SI tumor patients, exclusively, suggests that they are able to fulfill that unique role.

The potential of these markers becomes even more powerful when one considers that they may be measured together, as there are not only multiple proteins exclusively associated with a particular patient’s status but also multiple mRNAs and miRNAs. Measuring multiple markers together will always create a more accurate picture of the truth during diagnosis, leading to fewer false positives and negatives and to pinpointing the nature of the tumor and its progression. Because EVs can be isolated from blood, they are also very valuable as a diagnostic tool due to their collection and analysis being less invasive than biopsy. For this reason, blood plasma EVs, along with EVs collected from urine as described in Wang et al. (40), have the potential to greatly increase patient comfort and wellbeing during the diagnostic process. The first cancer diagnostic blood EV test became available commercially in 2016 (41). The ExoDx *Lung (ALK)* test measures both circulating tumor DNAs and RNAs to diagnose potential non-small-cell lung cancer patients.

Kim et al. (42) found pigment epithelium–derived factor (PEDF) proteins to be upregulated in EVs collected from the urine of PC patients. This is consistent with our finding that PEDF is overexpressed in OC and EC tumors. We also found that PEDF was underexpressed in SI patient blood EVs. The immunoglobulin-joining chain (IGJ) was found to be overexpressed in EVs from the urine of patients with extracapsular extension tumors (43). It appears that a similar story is true with EVs collected from the blood in which we found IGJ overexpressed in both OC and EC patients and the downregulation of IGJ in SI patients’ blood EVs. There are many other proteins from these urine EV studies that we did not detect in blood EVs. Although the proteins detected in this study are of EV origin, serum proteins such as complement factors and CRP might be adsorbed or stuck to the surface of EVs. In their examination of vesicular mRNAs in PC cell lines, Lázaro-Ibáñez et al. (36) found that LNCaP microvesicles contained an abundance of NKX3-1—a transcript we found overexpressed exclusively in healthy subjects and slightly downregulated in each progressing tumor type. Additionally, they found that LNCaP EVs contained high levels of TP53 and TMPRSS2 mRNAs, which we found abundant in the EVs of all tumor types and EC patients, respectively. We identified GSTP1 and ZNF185 as EV transcripts associated with healthy subjects and seminal vesicle–invading tumors, respectively, with CAV2 being associated with seminal vesicle invading tumors, and PES1 and CAMSAP1 being associated with healthy subjects. They identified these transcripts in the PC-3 cell line’s EVs, and, in both the PC-3 and LNCaP cell lines, they identified FASN and ETV1 transcripts, which they found to be strongly correlated to the progression of PC. This is consistent with our finding that these transcripts are found upregulated in the EVs of SI and EC patients, respectively.

STRING analysis reveals that several of the identified potential mRNA and protein biomarker candidates are known to interact with each other (**Supplementary Figure 1**). Because a cancer-specific panel was used for mRNA measurement, many of the mRNA candidates have known interactions with each other. Several of our best candidate protein biomarkers are known to interact with each other as well: ITIH3 has known interactions with KLKB1 and APOH. The network of protein interactions joins to the network of translated mRNA interactions *via* LBP, which is known to interact with VEGFA, and CRP, which, in addition to interacting with the protein FCN2, is coexpressed with APOH, APOA2, and LBP. Textmining for proteins that appear often in paper abstracts together suggests associations between CRP and the translated mRNAs of VEGFA, AKT1, CASP3, TP53, and B2M. Additionally, in a separate network, textmining reveals that CD5L, one of our top candidate protein biomarkers, is associated with the translated protein of IGJ, one of our top candidate mRNA biomarkers. Together, our top mRNA and protein candidates have known involvement in multiple cancer KEGG pathways, as well as platinum drug resistance and diabetes (**Supplementary Table 1**).

hsa-miR-141 has previously been identified in the blood plasma of PC patients as being correlated with the number of circulating tumor cells (44). Interestingly, several groups have reported that free plasma hsa-miR-141 is associated with ‘systemic’ or more advanced forms of PC (45, 46), yet, when we look into the EVs, we see that the highest expression levels by far are associated with patients with organ-confined tumors. hsa-miR-183 has previously been proposed as a potential biomarker for PC after observing its upregulation in tumor biopsies compared to BPH (47). We found hsa-miR-183 to be most associated with EC patient EVs, less with OC patient EVs, and least with SI patient EVs. hsa-miR-203 has previously been reported to be downregulated in metastatic PC—measured after miRNA isolation from LTL-313B- and LTL-313H-xenografted PC tissues in SCID mice (48). Saini et al. (49) also found that hsa-miR-203 is an “anti-metastatic” miRNA in PC and, when reintroduced to bone metastatic tumors, it inhibited several key elements of the metastatic cascade. In PC patient EVs, however, we see the opposite—namely, there is considerably more hsa-miR-203 in EC patients than OC and considerably more hsa-miR-203 in SI patients than in EC patients. EVs could potentially be the disposal mechanism the cancer cell uses to void itself of hsa-miR-203. hsa-miR-182 is known to aid PC in progression. Yao et al. (50) found that hsa-miR-182 was the single most upregulated miRNA in PC tissue. Hirata et al. (51) provided evidence that hsa-miR-182 promotes PC by targeting RECK, FOXF2, and MTSS1—tumor suppressor transcripts. Our data show hsa-miR-182 to be most differentially upregulated in the EVs of EC patients.

It is unclear at this point what biological reason there might be for these miRNAs’ presence in the EVs of PC patients. It may be reasonable to speculate, in light of the evidence for hsa-miR-23b-3p acting as a tumor suppressor in PC, that PC cells selectively load EVs with miRNAs as a means of disposing of it. An alternate possibility is that healthy cells load hsa-miR-23b-3p into EVs to be delivered to the tumor to suppress its growth as a mechanism of defense, but these possibilities require deeper exploration to really be considered.

We found previously that PCa EVs have the ability to transport nuclear receptors and other transcription factors directly to the nucleus of other cells (14). We have also suggested that this is potentially a mechanism by which PCa loses its sensitivity to androgen deprivation therapy. With this in mind, it is striking to observe that a large fraction of the proteins examined in this study are involved in localization and the establishment of localization and ask whether some large fraction of the EVs released into the blood by PCa are meant to deliver functional cargo to distal sites in the body. The exact biological role of each of these proteins—miRNAs and mRNAs—has not been characterized in this study, which sought to focus on markers, but is interesting and important for the understanding of EV tumor biology. A more extensive *in silico* follow-up, to characterize these functions, as well as the partial complementarities of the miRNAs involved could yield interesting and useful knowledge about why PCa cells selectively load this cargo into EVs or why healthy cells do in response to PCa. These data need to be verified *via* the processing of more patient samples using qRT-PCR for miRNAs and mRNAs and additional MS studies for proteins. It would also be valuable to compare the predictive efficacy of these potential biomarkers against the PSA blood test.

With the use of PC-specific mRNA and miRNA array panels, we wanted to concentrate on the RNA biomarkers that have a direct effect on PC pathogenesis, although many of the mRNA biomarkers such as TP53, CASP3, VEGFA, and BCL2 are associated with cancer in general rather than just PCa.

In conclusion, EVs collected from blood have the potential to be a less invasive, sensitive, and specific source of PC biomarkers that may have the potential to provide an accurate diagnosis for PC and assessing the disease stage.

## Data availability statement

The original contributions presented in the study are included in the article/**Supplementary Material**. Further inquiries can be directed to the corresponding author.

## Ethics statement

The studies involving human particioants were reviewed and approved by Ethics approval and consent to participate: This study is supported by ethical approval from the Hamilton Integrated Research Ethics Board (HIREB) #13-288-T. The study was performed in accordance with the Declaration of Helsinki. The patients/participants provided their written informed consent to participate in this study.

## Author contributions

Author Contributions: KA-N designed the project, supervised the study, analyzed the data, and wrote the manuscript. JC, SH-N. Prepared samples for proteomics analysis, and performed mRNA, miRNA arrays experiments, and helped in preparing the manuscript for publication. PM helped in preparing the manuscript for publication. All authors contributed to the article and approved the submitted version.
