# Supplementary material for: Characterization of proteins, mRNAs, and miRNAs of circulating extracellular vesicles from prostate cancer patients compared to healthy subjects
Source: Front Oncol. 2022 Dec 8;12:895555. doi: 10.3389/fonc.2022.895555 (PMC9776661; doi:10.3389/fonc.2022.895555)
Supplement: Supplementary file 1 [file Table_1.pdf]

Supplementary Table 1

KEGG Pathways

pathway	description	count in network	strength	false discovery rate
hsa05219	Bladder cancer	3 of 41	1.74	0.00047
hsa01524	Platinum drug resistance	5 of 70	1.73	4.97e-06
hsa05213	Endometrial cancer	4 of 57	1.72	5.80e-05
hsa04215	Apoptosis - multiple species	2 of 30	1.7	0.0072
hsa04115	p53 signaling pathway	4 of 72	1.62	0.00011
hsa05215	Prostate cancer	5 of 96	1.59	1.67e-05
hsa05210	Colorectal cancer	4 of 82	1.56	0.00016
hsa05223	Non-small cell lung cancer	3 of 68	1.52	0.0015
hsa05222	Small cell lung cancer	4 of 92	1.51	0.00019
hsa04979	Cholesterol metabolism	2 of 48	1.5	0.0156
hsa04933	AGE-RAGE signaling pathway in diabetic complications	4 of 98	1.49	0.00023
hsa05212	Pancreatic cancer	3 of 73	1.49	0.0018
hsa03320	PPAR signaling pathway	3 of 75	1.48	0.0018
hsa05418	Fluid shear stress and atherosclerosis	5 of 130	1.46	5.67e-05
hsa05145	Toxoplasmosis	4 of 105	1.46	0.00026
hsa01521	EGFR tyrosine kinase inhibitor resistance	3 of 78	1.46	0.0019
hsa04210	Apoptosis	5 of 132	1.45	5.67e-05
hsa05205	Proteoglycans in cancer	7 of 196	1.43	2.16e-06
hsa04510	Focal adhesion	7 of 198	1.42	2.16e-06
hsa04722	Neurotrophin signaling pathway	4 of 114	1.42	0.00033
hsa04370	VEGF signaling pathway	2 of 57	1.42	0.0202
hsa04071	Sphingolipid signaling pathway	4 of 116	1.41	0.00034
hsa01522	Endocrine resistance	3 of 95	1.38	0.0031
hsa05206	MicroRNAs in cancer	5 of 160	1.37	9.55e-05
hsa05211	Renal cell carcinoma	2 of 66	1.36	0.0256
hsa04664	Fc epsilon RI signaling pathway	2 of 66	1.36	0.0256
hsa05162	Measles	4 of 138	1.34	0.00059
hsa05230	Central carbon metabolism in cancer	2 of 69	1.34	0.0267
hsa04066	HIF-1 signaling pathway	3 of 106	1.33	0.0041
hsa05100	Bacterial invasion of epithelial cells	2 of 70	1.33	0.0269
hsa05218	Melanoma	2 of 72	1.32	0.0279
hsa05214	Glioma	2 of 72	1.32	0.0279
hsa05220	Chronic myeloid leukemia	2 of 75	1.3	0.0290
hsa05169	Epstein-Barr virus infection	5 of 193	1.29	0.00017
hsa05161	Hepatitis B	4 of 159	1.28	0.00096
hsa04919	Thyroid hormone signaling pathway	3 of 119	1.28	0.0054
hsa05152	Tuberculosis	4 of 168	1.25	0.0011
hsa05163	Human cytomegalovirus infection	5 of 218	1.24	0.00023
hsa04140	Autophagy - animal	3 of 130	1.24	0.0064
hsa04211	Longevity regulating pathway	2 of 87	1.24	0.0377
hsa05167	Kaposi sarcoma-associated herpesvirus infection	4 of 187	1.21	0.0015
hsa05226	Gastric cancer	3 of 144	1.2	0.0082
hsa05231	Choline metabolism in cancer	2 of 96	1.2	0.0447
hsa05170	Human immunodeficiency virus 1 infection	4 of 204	1.17	0.0018
hsa04660	T cell receptor signaling pathway	2 of 101	1.17	0.0483
hsa04620	Toll-like receptor signaling pathway	2 of 101	1.17	0.0483
hsa04064	NF-kappa B signaling pathway	2 of 101	1.17	0.0483
hsa05160	Hepatitis C	3 of 156	1.16	0.0100
hsa05131	Shigellosis	4 of 218	1.14	0.0022
hsa05164	Influenza A	3 of 165	1.14	0.0114
hsa05202	Transcriptional misregulation in cancer	3 of 171	1.12	0.0123
hsa04151	PI3K-Akt signaling pathway	6 of 350	1.11	0.00017
hsa05165	Human papillomavirus infection	5 of 325	1.06	0.00099
hsa04015	Rap1 signaling pathway	3 of 202	1.05	0.0188
