# Supplementary figures and images for: Characterization of proteins, mRNAs, and miRNAs of circulating extracellular vesicles from prostate cancer patients compared to healthy subjects
Source: Front Oncol. 2022 Dec 8;12:895555. doi: 10.3389/fonc.2022.895555 (PMC9776661; doi:10.3389/fonc.2022.895555)

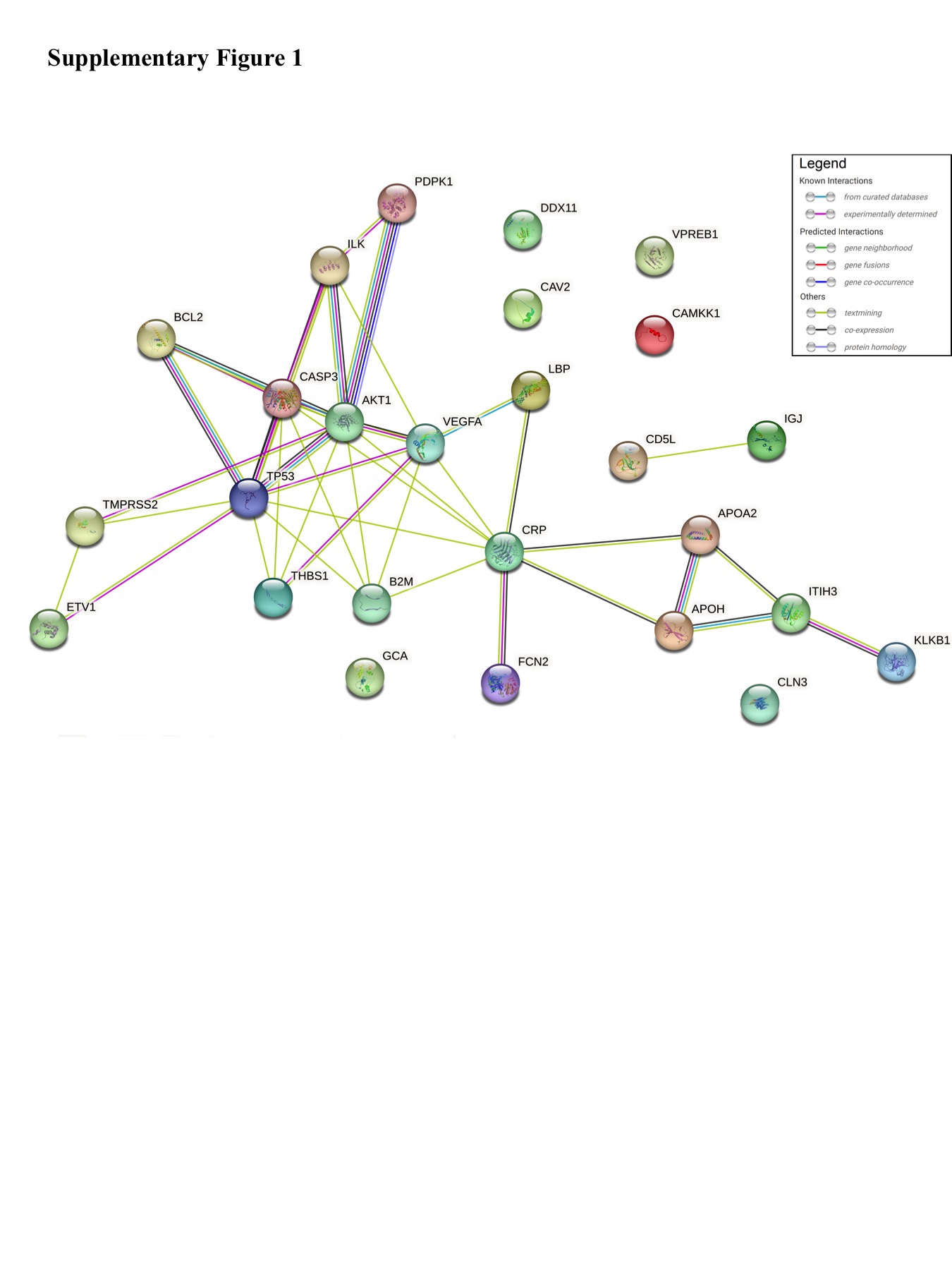

Supplement: Supplementary file 2 [file Image_1.jpg]
